# A Miniaturized Piezo Stack Impact Actuation Mechanism for Out-of-Plane Freely Moveable Masses

**DOI:** 10.3390/mi14061192

**Published:** 2023-06-03

**Authors:** Matthias C. Wapler, Constantin Peter, Koustav Kanjilal, Ulrike Wallrabe

**Affiliations:** 1 Laboratory for Microsystems Engineering for Medical Engineering, Faculty of Electrical Engineering and Information Technology, Otto-von-Guericke University Magdeburg, 39122 Magdeburg, Germany; matthias.wapler@ovgu.de; 2 Laboratory for Microactuators, Institute for Microsystems Technology—IMTEK, Albert-Ludwigs-Universität Freiburg, 79085 Freiburg, Germany; constantin.peter@uranus.uni-freiburg.de (C.P.); koustav0608@gmail.com (K.K.)

**Keywords:** piezo actuator, impact actuator, freely moveable masses

## Abstract

We present the prototype and analytical model of a miniaturized impact actuation mechanism, providing a fast out-of-plane displacement to accelerate objects against gravity, allowing for freely moving objects and hence for large displacements without the need for cantilevers. To achieve the necessary high speed, we chose a piezoelectric stack actuator driven by a high-current pulse generator, connected to a rigid support and a rigid three-point contact with the object. We describe this mechanism with a spring-mass model and compare various spheres with different masses and diameters and from different materials. As expected, we found that larger flight heights are achieved by harder spheres, achieving, e.g., approx. 3 mm displacement for a 3 mm steel sphere using a 3 × 3 × 2 mm^3^ piezo stack.

## 1. Introduction

In this study, we introduce a miniaturized impact actuation mechanism providing a fast and large out-of-plane displacement of freely moving objects without the need for cantilevers. With our 3 mm actuator dimensions, we bridge the gap between impact-type microactuators [1,2] in the sub-millimeter range and medium-sized actuators in the centimeter regime [3,4].

Cantilever-type beams and springs replace bearings in the microscopic world because they are compatible with microsystem technologies. They prevent movable structures from suffering from friction, which becomes large in relation to the other forces at small scales. Cantilever suspensions have been implemented in a plethora of microsystems for many different applications for in-plane, out-of-plane, and torsional movements. Popular application examples are acceleration sensors, gyroscopes, or scanning mirrors. Thereby, an object which is to be moved or displaced is always in a defined neutral position, as the force of the actuator needed for the displacement is balanced by the restoring force of the bent cantilever. Hence, cantilevers have the advantage of predictable forces and positions in addition to the absence of friction and wear with essentially no fatigue if made from silicon. Their disadvantages, however, are not much discussed. They are exactly the opposite of what we previously stated: the direction of motion is limited by the direction of the force of the actuator, we always have to overcome the restoring forces by an actuator which does not only need to accelerate the object but also balance the force of the cantilever springs. The cantilevers, therefore, limit the maximum displacement, and if we want to keep a displaced position constant we always have to provide some force by the actuator, which is constantly consuming power.

Only a few microactuators avoid cantilevers and perform phases of true free flight. One class is represented by magnetic levitation as has been shown, for example, by Badilita et al. [5], who combined two co-axial microcoils, one for levitation and one for stabilization, to inductively levitate a conductive disc. This concept was later combined with an electrostatic positioning mechanism [6]. The same holds true for freely floating spheres which were only recently introduced by Hoffmann et al., who used electrostatic bending actuators to transfer both force and torque to the spheres resulting in a step-wise rotation with multiple stable final positions [7].

Most microactuators are designed to provide steady force-displacement curves for a continuous motion, and their movable masses are usually suspended on cantilevers, as mentioned above. However, some impact microactuators have also been investigated, mostly to generate quasi-continuous motion. In the early years of MEMS, Pisano et al. [1] and Muller et al. [2] were among the first to build impact-based micro rotational [1] and linear [2] motors consisting of comb actuators that transfer their energy to a rotor, or a slider, respectively, creating a stepwise motion. The comb actuators were suspended to cantilevers, whereas the rotor was mounted on a central hub and the slider was completely free.

A special impact mechanism is used for so-called stick and slip microactuators which use a combination of inertia, on the one hand, and friction, on the other hand. Fujita et al. [8] presented a quasi-infinite linear displacement of a tiny silicon chip using electrostatic impact forces to let an inertial mass on this chip, being suspended to cantilevers, bump against a stopper. The chip underneath, however, was free to move, i.e., without a limiting suspension, and thus slipped stepwise across a surface. A comprehensive model of this system was presented by Nayfeh et al. [9], and Toshiyoshi et al. later extended this idea to motions in two dimensions [10]. A magnetically driven resonant impact actuator for haptic feedback in mobile phones has been demonstrated by Kwon et al. As this was slightly larger, the inertial mass was not suspended to microstructured cantilevers but to in-plane bendable steel springs instead [11].

A slightly different class of impact-based stick and slip actuators relies on alternating low and high-speed phases, for example, by slow elongation of an actuator loaded with a sliding mass and rapid contraction afterward, the first one dominated by high static friction, the second by lower dynamic friction. They have been implemented, e.g., by Hosaka et al. using piezoelectric actuation with in-plane polarization and superposing two longitudinal resonant modes, resulting in a close to saw-shaped step-wise displacement [12], or by Higuchi et al. using a shape memory alloy (SMA)-based actuation through rapid contraction of an SMA wire when resistively heated and slower cooling by surrounding air [13]. The same group even demonstrated actuation by laser-induced thermal [14] where the active part incorporated mirror surfaces for multiple laser beam reflections, thereby heating the element. As these impact-based stick and slip actuators are typically not in the microsystems domain, they are not suspended to cantilevers and provide untethered motion.

Another popular example of stick and slip microactuators, though not impact type and mentioned here for completeness, is the electrostatic scratch drive which was first introduced by Shono et al. [15] for in-plane movement and later extended by Fujita et al. for out-of-plane motion [16], and by Bright et al. even for the rotary motors [17]. Scratch drive actuators are also mostly suspended to cantilevers, however, with a different purpose, i.e., to suspend the scratch drive to a movable frame. In this case, the actuator does not need to balance the spring force.

**Figure 1:**
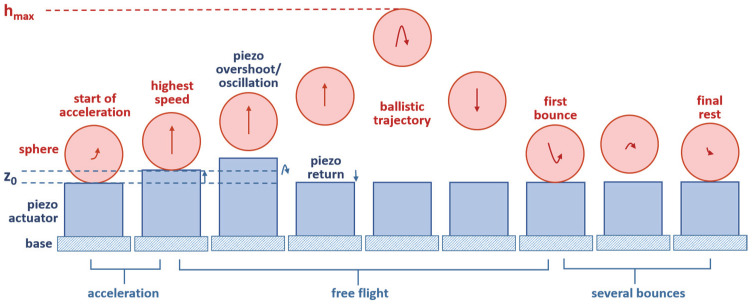
Actuation cycle of the ballistic sphere entering a free flight phase triggered by a short strong kick type actuation of a piezoelectric chip. The red arrows indicate qualitatively the speed, the blue arrows, however are not to scale (should be as long as the red ones). When falling down, the sphere experiences several bounces.

In order to further overcome the restrictions imposed by cantilevers, in this paper we study an out-of-plane impact “kick” actuation mechanism based on a tiny piezoelectric chip actuator. Our freely moving object is a sphere that performs a ballistic, i.e., free flight out-of-plane in one dimension according to gravitational acceleration. Falling down, the sphere exhibits several bounces until it comes to a standstill again. A single actuation cycle is illustrated in Figure 1.

We investigate the acceleration of spheres of various diameters and materials, i.e., with different masses and hardness. The setup is characterized by measuring those trajectories and obtaining the initial kinetic energy or speed as a function of the mass of the object and the displacement of the actuator. We then relate these results to a mechanical spring-mass model that takes into account the compliance of the actuator and the ballistic sphere in addition to their masses.

## 2. Experimental Setup

For a demonstrator, we used a small PZT stack actuator (3 × 3 × 2 mm^3^, Thorlabs PA3JEAW, Thorlabs, Inc., Newton, NJ, USA) with a fixed boundary condition at the bottom and an open boundary condition with three well-defined contact points for the projectile at the top. The ballistic sphere is then horizontally contained with a guidance structure that is supposed to guide the sphere back to the piezo in case it is released at an angle, but not to guide it in a vertical direction. We drove the piezo with a low-impedance pulse generator in order to achieve fast speeds.

### 2.1. Mechanical and Electric Setup

**Figure 2:**
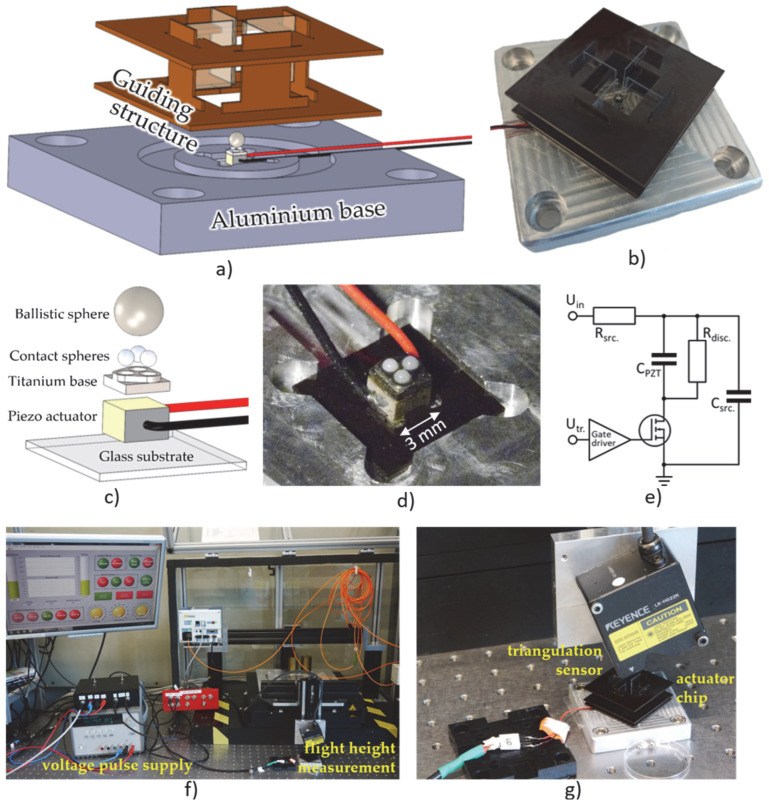
(**a**) Exploded view of the experimental setup; (**b**) photograph of the base plate, the guiding structures, and the projectile on the piezo actuator; (**c**) detail of the actuator with contact spheres from zirconia; (**d**) photograph of the piezo actuator (3 × 3 × 2 mm^3^) with contact spheres; (**e**) driving circuit; (**f**) overview on LabView controlled measurement setup including the voltage supply from (**e**); (**g**) close-up view of (**b**) below a triangulation sensor.

The mechanical setup is shown in Figure 2a–d. As a base, we used a 10 mm block of aluminum into which we glued a 10 × 10 mm sheet of 1 mm thick glass to provide a smooth surface and spread the load of the piezo, which we glued with low-viscosity epoxy (Araldite 2020) at the center.

To provide reliable contact to the ballistic sphere, we used three 1 mm contact spheres of zirconia. We glued them, again using Araldite 2020, in 1.1 mm holes with a 60° conical bottom and 1.35 mm pitch in a 1 mm thick titanium structure. To prevent the ballistic sphere from falling out of the experimental setup, we created a guidance structure that we laser-cut from 1 mm and 0.5 mm FR2 for the structural parts and 0.2 mm glass for the vertical guidance, leaving 0.125 mm space next to the sphere. We designed the structure to fix all its degrees of freedom when fitted together and fixated the pieces with cyanoacrylate “superglue”; it rests in a circular grove in the aluminum base.

To achieve a fast response time of the piezo leading to the desired kick and release of the sphere from the piezo, we needed a voltage source with a high peak current. For that purpose, we used the circuit shown in Figure 2e, where we stabilized the input voltage with a 2.2 µF capacitor C_src_ and applied it with an N-channel MOSFET (FQP32N20C) to the 113 nF piezo (C_PZT_).

### 2.2. Setup Characterization

**Figure 3:**
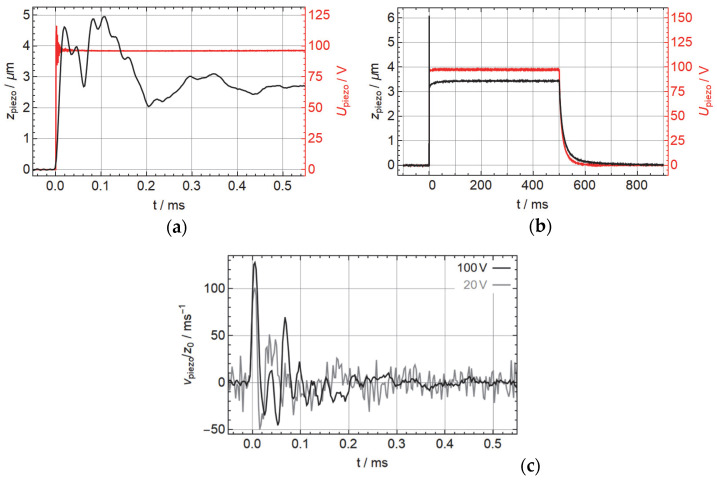
(**a**) Displacement of the surface of the actuator setup (black) and electric signal (red) for a trigger signal of 1 Hz and 50% duty cycle and an input voltage of 100 V; (**b**) initial response (left) and full cycle (right). (**c**) Corresponding vertical speed of the surface obtained through numerical differentiation, normalized by the steady-state displacement, for input voltages of 100 V and 20 V.

We characterized both the actuator and the flight trajectories of the ballistic spheres with optical sensors, in both cases taking a number of samples (21^2^ = 441 to 41^2^ = 1681) in a square raster over an area of 200 × 200 µm. While this gives a raster spacing of 10 or 5 µm, correspondingly, the relevance was to create a high number of data points for statistics to reduce the noise. For the characterization of the actuator, we used a Keyence LK-H022K (Keyence Corporation, Osaka, Japan) triangulation sensor that we operated in a LabView-controlled measurement set-up at a sampling frequency of 200 kHz; see Figure 2f,g. We show the mechanical and electrical response at 100 V input voltage in Figure 3 where we observe a fast mechanical response of approx. 10 µs, followed by a superposition of different resonances and a long-term creep. The height of the displacement should not be considered an exact value as it may be different at different positions on the surface. Due to the noise even with a large number of samples, it is difficult to compute an accurate value of the initial speed of the piezo surface. Taking samples at 20 V and 100 V, we find a maximum speed of approx. 100 to 130 $s^{-1}$ times the asymptotic displacement at the given voltage, which corresponds well to the response time. We also see that the electric response of the circuit, measured directly at the contact wires of the piezo, (red curves) is sufficiently fast in order not to affect the mechanical response (black curves). The difference of about 5% of the initial value compared to the input voltage can be explained by the ratio of the capacitances. We verified that the actuator is not limited by its internal resistance or inductance by measuring the impedance spectrum, which showed the typical resonance (480 kHz) and anti-resonance (600 kHz) in addition to a few highly suppressed modes that may result from the aluminum base. At the frequency range of relevance for the step response between 10 and 100 kHz, the actuator acts like a near-perfect capacitor with 104 nF and a phase between −89.2° and −89°. Between 20 and 24 kHz, there is a very small fluctuation in the phase of about 0.05°, and another one between 45 and 70 kHz with 0.15°, which may correspond to the mechanical ringing that we see in the step response in Figure 3.

**Figure 4:**
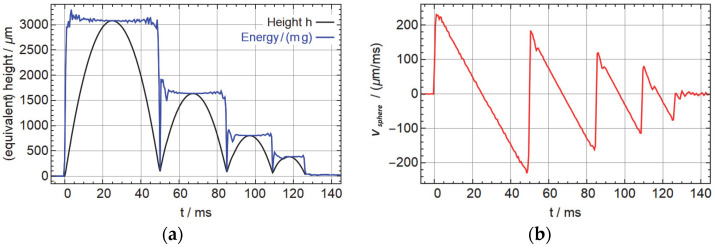
Single kick of a 3 mm hardened steel sphere, ejected at 100 V input voltage. (**a**) Vertical position and total computed energy (linear kinetic plus potential). (**b**) Vertical speed.

To demonstrate the kinematics, we show the trajectory of a 3 mm 1.4034 hardened steel sphere in Figure 4a that we measured with a chromatic confocal sensor (Polytec CL4, Polytec GmbH, Waldbronn, Germany) and the velocity as derived from the local slope in Figure 4b. The blue line in Figure 4a represents the equivalent height that represents the total energy composed of the potential and kinetic energy $h_{equiv}=\frac{E}{mg}=\frac{v^{2}}{2g}+h$. The height h was taken directly from the confocal sensor and the vertical speed v was obtained through numerical differentiation, which explains why the data is more noisy in the phases when the energy is dominated by the kinetic energy. We see the usual kinematics with approximately constant total energy and a series of bounces of the sphere on the piezo actuator. One has to keep in mind that the speed is comparatively noisy, and there may be contact with the guidance structure and a possible rotation of the sphere, in particular after the bounces. We did not track the actual spherical shape of the surface, so there may be some artifacts coming from a sideways motion in combination with the spherical surface profile that appears as an altered overall speed in combination with a slightly stronger gravitational acceleration. With a speed of at most approximately 0.2 m/s, we ignore aerodynamic friction for the rest of the paper.

### 2.3. Mechanical Model

**Figure 5:**
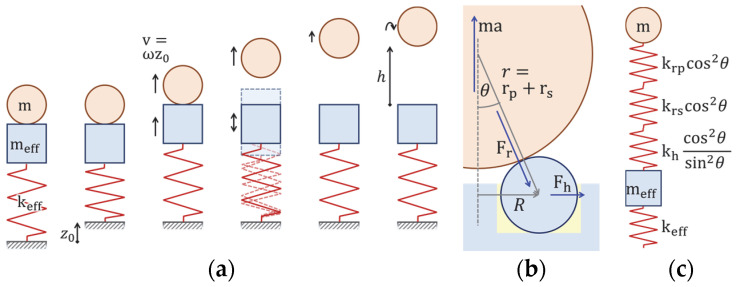
(**a**) Spring-mass model of the acceleration process. (**b**) Detailed model of the contact point between projectile and contact spheres. (**c**) Detailed spring-mass model.

To first order, such a device can be described by a simple spring-mass model with an effective spring constant $k_{eff}$ representing the stiffness of the actuator assembly including all effects caused by the glue and the geometry and possibly a section of the ballistic sphere, and a mass $M=m_{eff}+m$ consisting of an effective mass $m_{eff}$ of the actuator setup and the mass m of the sphere. For the sake of simplicity, we ignore damping in our model, knowing that it will not be exact, regardless of including damping. In Figure 5, we illustrate that immediately after applying the electric signal, the spring is in a compressed state corresponding to the total DC displacement $z_{0}$ (ignoring creep effects). It then relaxes with a harmonic motion with an angular frequency $\omega =\sqrt{k_{eff}/M}$, starting the sphere at the maximum speed $v=\omega z_{0}$. Hence, we expect a linear dependence of the initial speed of the sphere on the DC displacement of the actuator for different input voltages. The angular frequency should increase with decreasing mass, approaching the 100 to 130 m/s of the bare actuator setup at zero mass, even though $m_{eff}$ and $k_{eff}$ will depend on the mechanics of the actuator sphere contact, i.e., on the size of the sphere and its hardness.

We can describe those details to some approximation by introducing an additional spring constant *k_contact_* that describes the contact between the actuator assembly and the ballistic sphere. To obtain this spring constant, we assumed that the acceleration of the sphere is balanced by a force that is orthogonal to the surface at all three contact points, resulting in a horizontal force of the zirconia contact spheres acting on their titanium mount, and the glue as shown in Figure 5b,c. For this purpose, we consider the displacement of the center of the ballistic sphere, δz, if we apply a force *F* to it to first order using straightforward but careful geometry. In particular, we consider the spring constants *k_h_* describing the horizontal deformation or displacement of the contact spheres and *k_rs_* and *k_rp_* describing the radial deformation of the contact spheres and the ballistic spheres, respectively. $\frac{1}{k_{contact}}=\frac{\delta z}{F}$ becomes, then, for overall three contact points

(1) $$\frac{1}{k_{contact}}=\frac{3}{{cos}^{2}\theta }\left(\frac{1}{k_{rp}}+\frac{1}{k_{rs}}\right)+\frac{{sin}^{2}\theta }{{cos}^{2}\theta }\frac{3}{k_{h}}.$$

The first term results from the radial compression of the two spheres, and we see that this term is in a purely vertical load (θ=0) just the spring constant of the ballistic sphere and the contact spheres, and as θ approaches 90°, the overall spring constant vanishes, as on the one hand the radial component of the radial force diverges, and on the other hand the vertical displacement resulting from a radial compression diverges, too. The second term representing the horizontal displacement of the contact spheres in addition vanishes at (θ=0), where there is obviously no horizontal displacement of the contact spheres resulting from a vertical load. Splitting the deformation of the contact spheres and the ballistic spheres allows us to take into account the different deformations of the ballistic spheres, assuming, e.g., proportionality to the hardness of the material. We then further assume that the initial speed of the ballistic sphere is described by the slowest mode of the combined spring-mass system of $m_{eff}$ with displacement $z_{eff}$ that is coupled via $k_{eff}$ to an external constraint and via $k_{contact}$ to *m* with displacement *z.* This is described by the differential equation for coupled oscillators $m_{eff}{\ddot{z}}_{eff}+k_{eff}z_{eff}+\left(z_{eff}-z\right)k_{contact}=0$, $m\ddot{z}+\left(z-z_{eff}\right)k_{contact}=0$ with the solutions for the resonance frequency

(2) $$\omega^{2}=\frac{k_{contact}m_{eff}+k_{eff}\left(m+m_{eff}\right)\pm \sqrt{{\left(k_{contact}m_{eff}+k_{eff}\left(m+m_{eff}\right)\right)}^{2}-4{k_{eff}k}_{contact}m\;m_{eff}}}{2m\;m_{eff}}$$

of which we chose the negative sign to obtain the slower one of the two modes.

## 3. Measurements and Discussion

We demonstrated our setup using ballistic spheres of tungsten carbide, zirconia, and hardened and non-hardened stainless steel, summarized in Table 1, with diameters of 3, 4, and 5 mm.

### 3.1. Measurement Protocol

We measured the acceleration of each sphere over a sequence of input voltages from 20 to 100 V. For each combination, we measured a total of 441 events with a continuous trigger of 1 Hz on a 21 × 21 grid on 200 × 200 µm around the center of the sphere, again using the CL4 chromatic confocal sensor at a sampling rate of 2 kHz. To exclude effects from friction on the sidewalls of the guidance structure, surface defects in combination with different orientations of the ballistic spheres, dust particles, rotation of the sphere or improper contact with the actuator assembly, we placed several filters on the data of each shot, excluding data with:

- >0.5 ms deviation of the actual flight time from the expected flight time obtained from the maximum height;
- >1% or 2 µm/ms deviation of the initial speed from kinematic expectation;
- >4% deviation from the gravitational acceleration;
- A horizontal speed (obtained from the measured acceleration and sphere diameter) greater than 20 µm/ms or 15% of the vertical speed.

To be more robust against possible further errors, we then took the median value rather than the mean value of the remaining measurements. Hence, our results do not conclude how well all movements follow Newtonian kinematics, but they give the results of the median flight height for those that appeared to have a sufficiently vertical flight path with standard kinematics without friction.

To reference the flight height or the resulting initial speed to the piezo displacement, we measured the near-asymptotic displacement of the actuator as a function of the voltage. As in Section 2.2, we used our LK-H022K triangulation sensor, now at 20 kHz which is less noisy than the 200 kHz and only on an 8 × 8 grid over 200 × 200 µm. As it was difficult to measure on the ceramics spheres, we measured near the edge of the titanium plate, which may have a slightly different overall displacement. Using the same 1 Hz actuation frequency as for the acceleration of the spheres, we used averages over each of the last half of the rest state and displaced state to obtain the displacement. Finally, we fitted a 4th-order polynomial (without constant) into the voltage-dependent displacement.

### 3.2. Measurement Results

**Figure 6:**
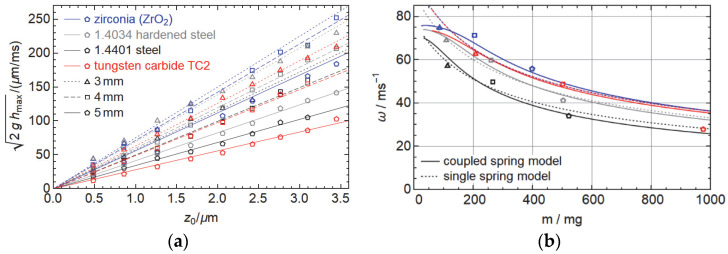
(**a**) Initial speed of the different projectiles as a function of the displacement of the piezo actuator. (**b**) Corresponding angular frequency of the spring-mass model as a function of the ballistic sphere mass. In both figures, the colors denote different materials and the shapes of symbols and dashings of lines denote the diameter of the spheres.

In Figure 6a, we show the median initial speed of the different ballistic spheres as a function of the piezo step height, obtained from the maximum flight height as measuring the speed is comparatively noisy. We find a very linear dependence of the initial velocity on the piezo displacement, as we expect from the spring-mass model, with slower velocities for the heavier spheres. In particular, for the smaller projectiles, we could not obtain data at all voltages; as for high voltages, some exceeded the measurements range of the sensor and the measurements became overall less reliable, probably due to a higher likelihood of friction with the guidance structure. In Figure 6b, we show the corresponding angular velocity as a function of the sphere mass for the different materials. As expected, we see a decreasing angular frequency with increasing mass and a convergence roughly towards the regime of the angular frequency that we measured for the unloaded actuator assembly in Section 2.2.

Fitting our model from Equations (1) and (2) to the data gives a reasonably good description of the behavior, but still with some deviations. It turned out that taking *k_rp_* in the model proportional to the hardness resembles the data much better than using Young’s modulus. There is, however little difference in which of the hardness values we use in the range in Table 1, and whether we use the given HRC values or the HV values (and convert the values for the steel spheres). Additionally, small modifications to the geometric model do not change the quality of the fit. One also has to take into account, however, that this is a 5-parameter model fitted to just 9 measurement values. Yet, the qualitative fit turns out to be much better than the simple spring-mass system with just $\omega =\sqrt{k_{eff}/\left({m+m}_{eff}\right)}$, even if we introduce an additional linear dependence of $m_{eff}$ and $1/k_{eff}$ on the hardness. Our fit predicts a rather low effective mass of just 16 mg and an effective spring constant of 0.11 N/µm compared to the mass of just the piezo of approx. 200 mg and 200 N/µm (typical values, [24]). Yet, those values correspond to a resonance frequency around 80 kHz, not far from the 100 to 130 kHz of the bare actuator assembly (see Figure 3c), so they are consistent and suggest that most of the deformation takes place near the top of the assembly. The value of *k_h_* is 0.27 or 0.33 N/µm depending on which hardness values we use, which makes sense given that the spheres are horizontally fixated by a glue layer. *k_rs_* and *k_rp_* combine to between 0.24 and 0.49 or 0.51 N/µm for the different materials.

**Figure 7:**
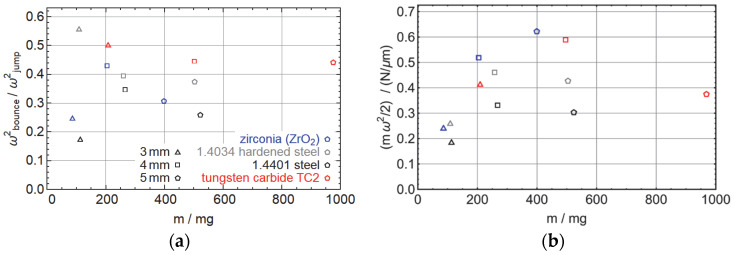
(**a**) Fraction of energy recovered in the first bounce for different objects. (**b**) Ratio of initial kinetic energy to the actuator displacement, which is, in an ideal simple spring-mass mode, the spring constant. Again, the colors denote different materials and the shapes of the symbols denote the diameter of the spheres.

In Figure 7a, we show the quality factor of the first bounce of the ballistic spheres, i.e., the ratio of the sphere energy in the initial acceleration phase and the first bounce obtained from the fit parameter ω. While the data is not perfect, we find by comparing ballistic spheres with similar mass, that tungsten carbide has the best energy recovery, followed by hardened steel, zirconia, and unhardened steel. This suggests that the energy recovery does depend on the material of the spheres, with a higher quality factor the harder the material and a higher quality factor for metals than ceramics, as zirconia has a similar hardness as tungsten carbide. Overall, this quality factor is hard to predict and depends on several factors that we ignored in our model: direct damping of the spring-mass model, friction between the ballistic spheres and the contact spheres due to horizontal displacement of the contact spheres during impact, and also the simply off-axis impact of the ballistic sphere.

To finally demonstrate how effectively our setup uses the possible strength of the actuator, we look in Figure 7b at the ratio of the energy of the spheres to the square of the actuator displacement, given by $\frac{1}{2}m\omega^{2}$. We see that the highest value is achieved for the 5 mm zirconia and 4 mm tungsten carbide spheres, with about 0.6 N/µm. Comparing this to the energy $\frac{1}{2}k$ that can be stored in the spring, we find that this value is, on the one hand, much larger than the fitted value for $k_{eff}$, casting a little doubt on our spring-mass system. The other values between 0.2 and 0.5 N/µm are not far off from the values of *k_h_*, *k_rs_,* and *k_rp_*. On the other hand, all of those values are still much smaller than the spring constant of a bare piezo, suggesting that our setup is not the most effective way to accelerate a sphere with a piezo actuator.

## 4. Conclusions

We have demonstrated a ballistic actuator based on a compact 3 × 3 × 2 mm^3^ piezo stack actuator, accelerating spheres with 3 to 5 mm diameter and different materials: untreated and hardened steel, ceramics (zirconia), and hard metal (tungsten carbide). To avoid effects due to uneven surfaces or dirt particles, we designed a contact structure consisting of three 1 mm zirconia spheres, mounted in a thin titanium structure. We found that this system can be reasonably well described by a spring-mass system describing the spring and mass of the lower part of the actuator assembly coupled to a spring of just the contact points and the mass of the ballistic sphere. Overall, this spring-mass model is generic to such setups if one adapts the spring constant of the contact points accordingly. According to our model, most of the elastic deformation takes place in the upper actuator assembly. This stresses that the design and fabrication of a contact structure that provides a well-defined contact but at the same time as little deformation as possible is the key to achieving a large momentum transfer to the spheres. Obviously, the height of the trajectories was larger, the lighter and harder the spheres; with the smallest zirconia spheres, we exceeded the 4 mm range of our measurement sensor.

The highest energy of the sphere per actuator displacement squared was approx. 0.6 N/µm, a factor of 130 smaller than what could be achieved with an ideal piezo actuator. Our results demonstrate that, while they allow for tightly packed arrays, these short-stroke actuators may not be the most effective approach to piezo ballistic actuators. For single actuators, it may be a more sensible approach to use bending or buckling actuators, such as [25,26], that potentially accelerate the spheres with a slower but larger stroke and are hence less sensitive to deformations of the contact points, but come at the price of a wider footprint. In fact, our results demonstrate that using chip actuators with an even smaller footprint is likely to provide results similar to our 3 mm actuators as the strength of the actuator itself is not the limiting factor, making the integration into arrays even more straightforward.

## Figures and Tables

**Table 1:** Summary of the materials.

Material	Density ^1^	Y-Modulus	Hardness
Zirconia (ZrO_2_)	6090 kg/m^3^	205 GPa ^4^	1200–1400 HV ^2^, 78 HRC ^3^
1.4034 hardened steel	7710 kg/m^3^	215 GPa ^5^	54–60 HRC ^2^, 60 HRC ^3^
1.4401 untreated steel	7990 kg/m^3^	200 GPa ^6^	25–39 HRC ^2,3^
Tungsten carbide TC2	14,800 kg/m^3^	669–696 GPa ^7^	1400–1600 HV ^2^, 78 HRC ^3^

^1^ Measured, ^2^ supplier data [18], ^3^ supplier data [19], ^4^ supplier data [20], ^5^ supplier data [21], ^6^ supplier data [22], ^7^ Matweb [23].

## Data Availability

The data presented in this study are available on request from the corresponding author.
